# Exploring the DNA Methylation Profile of Genes Associated with Bladder Cancer in Bladder Tissue of Patients with Neurogenic Lower Urinary Tract Dysfunction

**DOI:** 10.3390/ijms25115660

**Published:** 2024-05-23

**Authors:** Periklis Koukourikis, Maria Papaioannou, Stavroula Pervana, Apostolos Apostolidis

**Affiliations:** 1 2nd Department of Urology, Aristotle University of Thessaloniki, General Hospital ‘Papageorgiou’, 56403 Thessaloniki, Greece; periskouk@gmail.com; 2 Department of Biological Chemistry, Medical School, Aristotle University of Thessaloniki, 54124 Thessaloniki, Greece; mpapaioannou@auth.gr; 3 Department of Pathology, General Hospital ‘Papageorgiou’, 56403 Thessaloniki, Greece; st.pervana@gmail.com

**Keywords:** DNA methylation, epigenetics, neurogenic lower urinary tract dysfunction, neurogenic bladder, inflammation

## Abstract

DNA methylation is an epigenetic process that commonly occurs in genes’ promoters and results in the transcriptional silencing of genes. DNA methylation is a frequent event in bladder cancer, participating in tumor initiation and progression. Bladder cancer is a major health issue in patients suffering from neurogenic lower urinary tract dysfunction (NLUTD), although the pathogenetic mechanisms of the disease remain unclear. In this population, bladder cancer is characterized by aggressive histopathology, advanced stage during diagnosis, and high mortality rates. To assess the DNA methylation profiles of five genes’ promoters previously known to be associated with bladder cancer in bladder tissue of NLUTD patients, we conducted a prospective study recruiting NLUTD patients from the neuro-urology unit of a public teaching hospital. Cystoscopy combined with biopsy for bladder cancer screening was performed in all patients following written informed consent being obtained. Quantitative methylation-specific PCR was used to determine the methylation status of RASSF1, RARβ, DAPK, hTERT, and APC genes’ promoters in bladder tissue samples. Twenty-four patients suffering from mixed NLUTD etiology for a median duration of 10 (IQR: 12) years were recruited in this study. DNA hypermethylation was detected in at least one gene of the panel in all tissue samples. RAR-β was hypermethylated in 91.7% samples, RASSF and DAPK were hypermethylated in 83.3% samples, APC 37.5% samples, and TERT in none of the tissue samples. In 45.8% of the samples, three genes of the panel were hypermethylated, in 29.2% four genes were hypermethylated, and in 16.7% and in 8.3% of the samples, two and one gene were hypermethylated, respectively. The number of hypermethylated genes of the panel was significantly associated with recurrent UTIs (*p* = 0.0048). No other significant association was found between DNA hypermethylation or the number of hypermethylated genes and the clinical characteristics of the patients. Histopathological findings were normal in 8.3% of patients, while chronic inflammation was found in 83.3% of patients and squamous cell metaplasia in 16.7% of patients. In this study, we observed high rates of DNA hypermethylation of genes associated with bladder cancer in NLUTD patients, suggesting an epigenetic field effect and possible risk of bladder cancer development. Recurrent UTIs seem to be associated with increased DNA hypermethylation. Further research is needed to evaluate the impact of recurrent UTIs and chronic inflammation in DNA hypermethylation and bladder cancer etiopathogenesis in NLUTD patients.

## 1. Introduction

DNA methylation is an epigenetic mechanism that was first described by Hotchkiss in 1948, and it occurs due to the addition of a methyl group to the 5th carbon atom of cytosine, predominately when cytosine precedes a guanidine nucleotide (CpG dinucleotide). This process forms 5-methylcytosine and is catalyzed by the DNA methyltransferases enzymes (DNMT1, DNMT3A, and DNMT3B) [1]. Rich areas of CpG dinucleotides, called CpG islands, are usually found in genes’ promoters, which are mainly unmethylated to allow for gene transcription. Methylation of these CpG islands results in transcriptional suppression. In general, DNA methylation is a regulator of gene expression and has a vital role in several processes such as gene imprinting in normal development, repetitive DNA silencing, and X chromosome inactivation in females [2]. Its dysregulation has been associated with various human diseases, including autoimmune conditions, cardiovascular disorders, neurodegenerative disorders, and cancer [3].

Aberrant DNA methylation is a common event in bladder cancer, occurring in 50–90% of cases. DNA hypermethylation has been detected in the promoter sites of several genes such as CDH4, PAX5A, HOXA9, MEIS1, VIM, CDKN2A, RASSF1, MSH6, MGMT, TERT, GATA2, DAPK, CCND2, and GSTP1 [4]. These genes are involved in various molecular processes, including cell cycle control, apoptosis, DNA repair, tumor suppression, cell invasion, and other regulatory pathways. Transcriptional silencing of these crucial genes via hypermethylation mainly occurring in the promoter region contributes to the development and progression of bladder cancer [5].

The lower urinary tract (LUT), comprising the bladder and the bladder outlet (bladder neck, the urethra, the urethral sphincter, and the pelvic floor), serves the dual roles of storing and intermittently expelling urine. The normal function of the LUT requires a complex and well-coordinated neural control at multiple levels. Neurogenic lower urinary tract dysfunction (NLUTD), historically termed “neurogenic” bladder, arises as a consequence of a variety neurological disorders that affect the coordination of the LUT, leading to symptoms and complications that significantly impact a patient’s quality of life [6]. Advantages in the treatment and follow up of these patients and improvements in the diagnosis and management of NLUTD have reduced the complication rates and mortality resulting from renal causes and have resulted in an increased life expectancy for the patients. As a consequence of the extended lifespans, individuals with neurological conditions inevitably encounter risk factors similar to the general population for developing other significant diseases, including bladder cancer [7].

Additional risk factors for bladder cancer beyond those identified in the general population have been proposed for individuals with NLUTD. Initially, the use of an indwelling catheter for bladder management was identified as a significant risk factor [8]. Groah et al. have, in their cohort, identified that the indwelling catheter serves as an independent risk factor for bladder cancer development in spinal cord injury (SCI) patients, demonstrating a time-dependent association [9]. Furthermore, two other potential causes of chronic bladder inflammation, recurrent urinary tract infections and bladder stones, are both highly prevalent in NLUTD patients and have been also reported as risk factors for bladder cancer [8,10].

Recent studies suggest that bladder cancer is not as frequent as previously reported in NLUTD patients, with an incidence range from 0.2% to 2% [11]. However, in these individuals, bladder cancer tends to be diagnosed at an earlier age and at a more advanced disease stage, characterized by a notable prevalence of muscle-invasive tumors and a high occurrence of squamous cell carcinomas. Consequently, these neoplasms exhibit a worse prognosis, and NLUTD patients with bladder cancer experience an approximately sevenfold-higher risk of mortality compared to the general population [12]. Despite these data, there is only limited research concerning the underlying mechanisms of bladder carcinogenesis in this specific population. In our previous study, we observed more frequent DNA hypermethylation in a panel of five genes in the urine of NLUTD patients’ compared with controls [13].

In this study, our objective was to explore the DNA hypermethylation of a panel of five genes’ promoters in the bladder tissue of NLUTD patients {Ras-Association domain Family member 1 (RASSF1), Retinoid Acid Receptor beta (RARβ), Death-Associated Protein Kinase (DAPK), Telomerase Reverse Transcriptase (TERT), and Adenomatous Polyposis Coli (APC)}. These specific genes have well-documented roles as tumor suppressors and have been strongly associated with bladder cancer in the existing literature [14,15,16,17].

## 2. Results

This study included twenty-four patients (11 women and 13 men) of mixed NLUTD etiology. The majority of the patients were suffering from multiple sclerosis (n = 9/37.5%) and the second most common NLUTD etiology was spinal cord injury or disease (n = 7/29.2%). Most patients were using clean intermittent catheterization (CIC) (n = 18/75%) to empty their bladders, four (16.7%) had spontaneous urination, and two (8.3%) had an indwelling catheter. The patients’ demographics and characteristics are summarized in Table 1.

**Figure 1:**
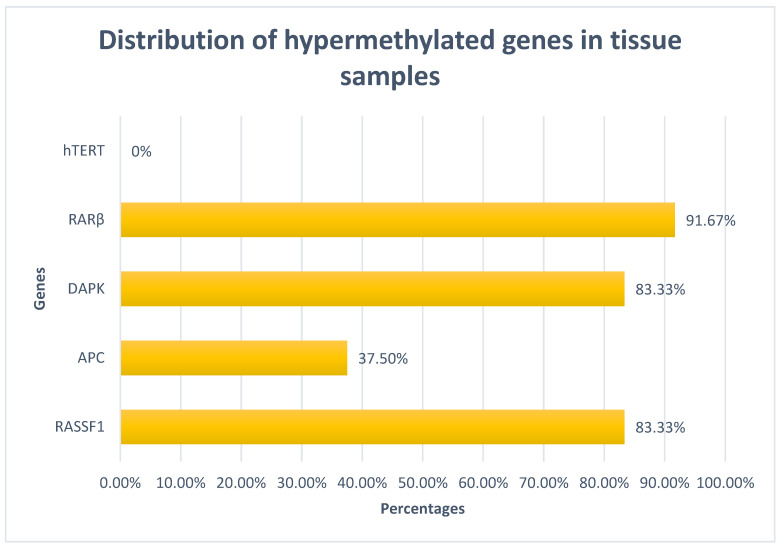
Distribution of hypermethylated genes of the panel.

DNA was successfully extracted from all the tissue samples. The panel of genes was positive for hypermethylation in all the tissue samples. RAR-β was hypermethylated in 22/24 (91.7%) samples, RASSF and DAPK were hypermethylated in 20/24 (83.3%) samples, and APC in 9/24 (37.5%) samples. TERT was not found to be hypermethylated in any of the tissue samples (Figure 1). Furthermore, three genes of the panel were hypermethylated in 11/24 (45.8%) tissue samples, four genes were hypermethylated in 7/24 (29.2%) tissue samples, two and one genes were hypermethylated in 4/24 (16.7%) and 2/24 (8.3%) of the tissue samples, respectively. There was no statistically significant correlation between hypermethylation of a particular gene and factors such as age, gender, body mass index (BMI), smoking in packyears, duration of NLUTD, duration of catheter usage, or history of recurrent urinary tract infections (UTIs) (*p* > 0.05). A significant association was identified between the number of hypermethylated genes of the panel and recurrent UTIs (*p* = 0.0048) (Table 2). No other significant association was found between the number of hypermethylated genes and age, gender, BMI, smoking in packyears, duration of NLUTD, or duration of catheter usage (*p* > 0.05).

The cystoscopy findings were normal in 16 patients, while in 8 patients the results were recorded as suspicious. The histopathology results of biopsies were normal in 2 patients (8.3%), while chronic inflammation was found in 2 patients (83.3%) (mild n = 7, moderate n = 9, severe = 4), and squamous cell metaplasia in 4 patients (16.7%). The cytology results were negative in all patients.

## 3. Discussion

In this study, we observed a high rate of hypermethylation in a panel of five genes (RASSF1, RARβ, DAPK, hTERT, and APC). All the patients in our study exhibited hypermethylation in at least one gene from the panel, with nearly half of them found to have three genes hypermethylated.

The genes in our panel that were found to be hypermethylated are considered to be tumor suppressors, pivotal in regulating diverse cellular functions. RASSF1 is an extensively researched gene known for its tumor suppressor function, engaging in the control of cell growth, modulation of the cell cycle, and initiation of programmed cell death [18]. RARβ, in response to retinoic acid binding, can activate or repress the expression of target genes involved in various cellular processes, including cell cycle regulation, cell differentiation, proliferation, and apoptosis [19]. The DAPK gene, which encodes a serine/threonine kinase, is an inducer of cell death in response to different stimuli, including stress, DNA damage, and cytokines [20]. APC is essential in modulating cell growth and division, primarily by controlling the Wnt signaling pathway [21]. Previous studies have demonstrated a significant correlation between dysregulation/inactivation of these genes through promoter hypermethylation and bladder cancer risk [14,15,16,17].

The TERT gene encodes the catalytic component of telomerase responsible for its activity. Telomerase functions by adding repetitive DNA sequences onto chromosome ends, thus counteracting the natural shortening process that occurs during cell division and maintaining telomere length [22]. TERT promoter hypermethylation, a common finding in cancerous tissues and urothelial carcinomas expressing TERT, offers a distinct pathway compared to the conventional suppression of gene expression [23]. The absence of hypermethylation detection in the promoter region of the TERT gene in our study might be due to the absence of malignant tissue in our samples, as it is documented that TERT induction and telomerase activation occur during the later stages of carcinogenesis [24]. Furthermore, a previous study in a Greek population revealed a TERT hypermethylation rate of 2.2% in patients with bladder cancer, supporting the observation that variations in DNA methylation exist among different human populations across various macro- and micro-geographical scales [25,26].

In this study population, suspicious flat mucosa lesions were prevalent on cystoscopy, appearing in approximately one-third of the patients. Notably, no bladder tumors were identified upon cystoscopic examination. As previously reported, cystoscopy is challenging in NLUTD patients, with low sensitivity and specificity for bladder cancer diagnosis [27]. This underscores the diagnostic difficulties and possible need for more bladder biopsies, which is common practice in our institution. While histopathological examination did not reveal the presence of bladder cancer, most of our patients exhibited abnormal histological findings. The high incidence of chronic inflammation observed in our study closely resembled findings from a previous investigation involving NLUTD patients with suprapubic indwelling catheters [28]. However, only two individuals in our study group were on indwelling catheters. This suggests that chronic bladder inflammation in the NLUTD population cannot be solely attributed to the presence of a permanent catheter.

Keratinizing squamous metaplasia of the bladder, a rare finding in the general population, was also observed in four patients of the study group. Keratinizing metaplasia typically develops in conditions characterized by chronic inflammation, such as prolonged use of indwelling catheters, the presence of bladder stones, diverticula, schistosomiasis infection, and neurogenic bladder dysfunction. This condition is considered to be a pre-cancerous lesion and has been linked to the onset of squamous cell carcinoma, with a variable latency period ranging from 4 to 28 years [29]. However, the risk of carcinoma development is controversial, especially in the absence of dysplasia, which was the rule in our patients [30,31]. Regular follow up, which includes cystoscopy and biopsy, is mandatory. Recently, a case report described the effectiveness of intravesical therapy with hyaluronic acid in resolving squamous metaplasia in a patient without a neurogenic disease [32].

The detection of DNA hypermethylation in genes previously associated with bladder cancer in the non-cancerous tissue in all of the patients of this study may indicate the existence of an epigenetic “field defect”, which has been previously described in bladder cancer by Wolff et al. [33]. In their study, normal-appearing tissue samples obtained at least 5 cm apart from a bladder invasive tumor had 169 hypermethylated loci, 89% of which were also detected in the invasive tumor. The authors suggested the presence of an epigenetic “field defect”, meaning that the hypermethylation was present in normal cells prior to the initiation of carcinogenesis [33]. Therefore, our findings suggest that DNA hypermethylation may represent an early event in the carcinogenesis pathway preceding the development of bladder cancer in the NLUTD population. Data from patients with NLUTD due to spinal cord injury showed that the median latency period between injury and bladder cancer diagnosis ranges from 18 to 34 years [12]. Similarly, in chronic lymphocytic leukemia, studies have indicated the detection of cancer-specific DNA hypermethylation in blood samples received over a decade prior to the diagnosis of the disease [34].

Several mechanisms could have possibly led to the high rates of hypermethylation in our NLUTD population. Pathogens, like bacteria and viruses, could induce aberrant DNA methylation. For example, the Epstein–Barr virus and the hepatitis B virus protein X have been identified as direct inducers of abnormal DNA methylation through the dysregulation of DNMTs [35,36]. It is known from the literature that NLUTD patients have a high prevalence of asymptomatic bacteriuria and an increased incidence of recurrent UTIs [37]. Indeed, the majority of our patients (58.3%) suffered from recurrent UTIs. Furthermore, in the general population, recurrent UTIs have been associated with elevated bladder cancer risk, although the pathogenesis mechanisms remain unclear [38]. In in vitro studies, urinary tract infection induced by Escherichia Coli resulted in increased DNMT1 activity, DNA methylation, and downregulation of the tumor suppressor gene CDKN2A [39]. In our analysis, recurrent UTIs were associated with the number of hypermethylated genes of the panel. More specifically, all the patients found to have four genes hypermethylated had recurrent UTIs. Consequently, DNA hypermethylation associated with recurrent UTIs may potentially influence the process of bladder carcinogenesis.

Chronic inflammation, a very common finding in our bladder biopsies, may also contribute to the elevated incidence of DNA hypermethylation observed in our study. Evidence supports the idea that inflammatory molecules, such as cytokines, chemokines, and reactive oxygen species, can activate the signaling pathways that regulate the DNA methylation processes. For example, nuclear factor-kappa B signaling, a key mediator of inflammation, can directly regulate the expression of DNMTs, leading to aberrant DNA methylation. Through these mechanisms, chronic inflammation could induce DNA methylation changes within cells, thereby contributing to tumor development, especially during the initiation phase of carcinogenesis [40,41]. Indeed, approximately 20% of cancers worldwide are related to infection and chronic inflammation [42].

Particularly for bladder cancer, the association of chronic inflammation with bladder carcinogenesis is well-documented in urinary Schistosomiasis. Chronic inflammation in the bladder caused by the parasitic infection from Schistosoma haematobium has been strongly connected with the occurrence of bladder cancer. Histologically, Schistosoma-associated bladder cancer is characterized by a high incidence of squamous cell carcinoma and muscle-invasive disease [43]. Thus, bladder cancer caused by schistosomiasis and bladder cancer in NLUTD population appear to have similar features concerning histological subtypes and aggressiveness. Although the majority of data on hypermethylation focus on the urothelial subtype of bladder carcinoma, the published literature also supports associations of the genes in our panel with squamous cell carcinoma, particularly in the context of schistosomiasis. DAPK, APC, RASSF1, and RARβ are among the 12 genes that were frequently found to be hypermethylated in bladder specimens of squamous cell histology. It is noteworthy that Schistosoma-associated cases demonstrate a greater number of hypermethylated genes compared to those not associated with the infection [44].

Carcinogenesis is a multistage process influenced by various factors, including epigenetic alterations, environmental carcinogens, and genetic factors. Our findings of the high incidence of DNA hypermethylation in tumor suppressor genes associated with bladder cancer do not conclusively indicate that all the patients will develop bladder cancer. DNA hypermethylation could be just one hit in the multiple-hit carcinogenesis theory. Hence, these individuals may not be exposed to other carcinogens or may not have genetic predisposition, all of which are factors needed to finally trigger the malignant transformation of bladder tissue. However, as these patients presumably have an elevated risk of invasive bladder cancer development, preventative measures, for example smoking cessation and regular follow-up, should be applied in order to reduce the risk or diagnose the disease at an early stage.

While this study provides a novel insight into the DNA methylation patterns in the bladder tissue of NLUTD patients, some limitations should be acknowledged. First, the small sample size of 24 patients may have limited our intention to explore associations between DNA methylation and patients’ characteristics. Additionally, the cross-sectional design of our study prevents us from establishing causal relationships between DNA methylation and bladder cancer development. Longitudinal cohort studies are needed to validate our findings and investigate this possible causal relationship. Lastly, our study focused on only five genes associated with bladder cancer, potentially overlooking other important genes or pathways involved in the pathogenesis of bladder cancer in NLUTD patients.

## 4. Materials and Methods

### 4.1. Study Population

This was a prospective, cross-sectional study conducted at the neuro-urology outpatients clinic of the urology department of a public teaching hospital. The study protocol was approved by the local university Ethics Committee and registered in the International Traditional Medicine Clinical Trial Registry (ISRCTN37551129). All participants signed written informed consent before their recruitment in the study. Consecutive patients suffering from NLUTD for at least 5 years and scheduled for cystoscopy with cold cup biopsy and wash cytology for bladder cancer screening were asked to participate in this study. Exclusion criteria included age under 18 years, prior diagnosis and treatment of bladder cancer, or other malignancy of the urinary tract. Patients’ demographics, data on the etiology and NLUTD duration, method of voiding, catheter usage duration, history of hematuria, recurrent urinary tract infections, and bladder stones were collected.

### 4.2. Cystoscopy

All patients underwent rigid cystoscopy under local anesthesia with at least four cold-cup biopsies from specific bladder regions (sidewalls, posterior wall, dome) and biopsies from areas of suspicion. All the procedures were performed by a single board-certified urologist. Findings were categorized as normal, suspicious in case of flat mucosa bladder lesions, or indicative of bladder tumor.

### 4.3. Urine Cytology

Bladder irrigation through the cystoscope was carried out during cystoscopy. The specimens were processed in the cytology laboratory of the hospital. Cytopathologists were blinded to the patients’ cystoscopy results. Positivity in cytology was defined by the identification of suspicious or malignant cells.

### 4.4. Tissue Sample Processing, DNA Isolation, Methylation-Specific PCR

The tissue samples for DNA methylation analysis were snap-frozen in liquid nitrogen and stored at −80 °C until processing. Frozen tissues underwent processing using liquid nitrogen and were pulverized utilizing mechanical techniques. DNA was extracted from the tissue samples using the Cells and Tissue DNA Isolation Kit (Norgen Biotek Corp., Thorold, ON, Canada). Extracted DNA underwent bisulfite conversion using the EZ DNA Methylation-GoldKit (Zymo Research, Irvine, CA, USA), according to the manufacturer’s instructions. Converted DNA was mixed with methylation-specific primers, Taqman probes, a premixed solution of nucleotides, and DNA polymerase (Luna Universal Probe qPCR Master Mix, New England Biolabs, Ipswich, MA, USA). The primers and the probe were constructed according to previous methylation studies [45,46] by IDT (Integrated DNA Technologies, Coralville, IA, USA) to specifically amplify the bisulphite-converted promoter of the gene of interest, and their sequences are listed in Table 3. Quantitative methylation-specific PCR (qMSP) was performed to detect bisulfite-induced changes at the promoters of the genes, RASSF1, RARβ, DAPK, TERT, and APC, using the Applied Biosystems StepOnePlus Real Time PCR System (Thermo Fisher Scientific, Inc., Waltham, MA, USA). For the quantification of methylation, the housekeeping gene ACTB was used for each PCR reaction and the ddCt formula was applied according to the literature [47]. Fully methylated and unmethylated DNA (Epitect Control DNA Set, Qiagen, Hilden, Germany) were used as positive and negative controls, respectively. All reactions were run in triplicate. The panel of genes was recorded as positive if hypermethylation was detected in the promoter region of at least one of the five genes.

### 4.5. Histopathology

The biopsy samples were processed in the Hospital’s Pathology Laboratory, stained with hematoxylin–eosin, and examined by a single uropathologist. The results were reported according to the WHO 2004/2016 histological classification for urothelial carcinomas and flat lesions [48].

### 4.6. Statistical Analysis

The primary outcome of this study was to explore the methylation status of our panel in the bladder tissue of NLUTD patients. The secondary outcomes were associations between the methylation status of each gene and the number of hypermethylated genes in the panel with the demographics and the clinical characteristics of the patients.

Descriptive statistics were estimated for each variable. The frequency and relative frequency were reported for categorical variables. The data from quantitative variables were summarized by measures of central tendency (mean or median) and variation {(standard deviation (SD) or interquartile range (IQR)} depending on their distribution; the Shapiro–Wilk test used for assessing the normality of data. Bivariate analysis tests (Fisher’s exact test, Wilcoxon rank sum test, Spearman’s rank correlation coefficient) were used to explore associations between meaningful patients’ variables and panel hypermethylation. For all statistical analyses, a two-tailed *p*-value < 0.05 was defined as statically significant. All statistical analyses were performed using the statistical package R (The R Project for Statistical Computing, Version 3.4.4 for Windows) and R studio (Version 1.1.453 for Windows).

## 5. Conclusions

In this study exploring the DNA methylation profile in the bladder tissue of NLUTD patients, we observed high rates of DNA hypermethylation of bladder cancer-associated genes, suggesting an epigenetic field effect and a possible risk of bladder cancer development later in life. Recurrent UTIs seem to be associated with increased DNA hypermethylation in the bladder. Further research is needed to elucidate the complex interplay between recurrent UTIs, chronic inflammation, and DNA methylation in bladder carcinogenesis specifically in NLUTD patients. This is a critical step for advancing our understanding of the disease mechanisms and developing biomarkers and strategies for prevention, early diagnosis, and treatment of the disease.

## Figures and Tables

**Table 1:** Patients’ demographics and clinical characteristics.

Variable	n = 24
Age, years (SD)	44.3 (14.7)
Gender, n (%) - Male - Female	
	13 (54.2%)
	11 (45.8%)
BMI, kg/m^2^ median (SD)	24.1 (4.2)
Smoking in packyears, median (IQR)	13 (23.5)
Neurological disease, n (%) - Spinal cord injury/disease - Multiple sclerosis (MS) - Meningomyelocele - Other	
	7 (29.2)
	9 (37.5)
	4 (16.7)
	4 (16.7)
NLUTD duration, years median (IQR)	10.0 (12.0)
Method of voiding, n (%) - CIC - IDC [urethral or suprapubic] - Spontaneous	
	18 (75.0)
	2 (8.3)
	4 (16.7)
Catheter usage duration (CIC or IDC),	
years (SD)	7.2 (5.5)
History of hematuria, n (%)	
Yes	1 (4.2)
No	23 (95.8)
Recurrent UTIs, n (%)	
Yes	14 (58.3)
No	10 (41.7)
History of hematuria, n (%)	
Yes	1 (4.2)
No	23 (95.8)

**Table 2:** Association of number of hypermethylated genes of the panel with clinical characteristics of the patients.

Variable/Number of Methylated Genes	1 (n = 2)	2 (n = 4)	3 (n = 11)	4 (n = 7)	*p*-Value
Age	40 (37, 42)	54 (50, 58)	39 (24, 58)	43 (40, 48)	0.729
BMI	23.3 (22.7, 23.9)	20.9 (20.6, 21.4)	24.7 (21.3, 26.1)	25.7 (23.9, 26.2)	0.074
Packyears	22 (21, 23)	30 (23, 35)	10 (1, 22)	8 (2, 15)	0.113
Gender					0.403
Female	1 (50%)	2 (50%)	3 (27%)	5 (71%)	
Male	1 (50%)	2 (50%)	8 (73%)	2 (29%)	
Recurrent UTIs	1 (50%)	0 (0%)	**6 (55%)**	**7 (100%)**	**0.0048**
NLUTD/years	24 (15, 34)	6 (6, 8)	10 (8, 18)	15 (12, 25)	0.502
Catheter use/years	5.0 (2.5, 7.5)	2.0 (1.5, 2.8)	9.0 (5.5, 13.0)	6.0 (4.0, 11.5)	0.107

**Table 3:** The sequences of primers and probes for the quantitative methylation specific real-time PCR.

Gene	Primer/Probe	Sequence
APC [45]	Forward	5′-GAACCAAAACGCTCCCCAT-3′
	Reverse	5′-TTATATGTCGGTTACGTGCGTTTATAT-3′
	Probe	5′-/56-FAM/CCCGTCGAA/ZEN/AACCCGCCGATTA/31ABkFQ/3′
DAPK [46]	Forward	5′-TCGTCGTCGTTTCGGTTAGTT-3′
	Reverse	5′-TCCCTCCGAAACGCTATCG-3′
	Probe	5′-/56-FAM/CGACCATAA/ZEN/ACGCCAACGCCG/31ABkFQ/3′
RARβ [45]	Forward	5′-GGGATTAGAATTTTTTATGCGAGTTGT-3′
	Reverse	5′-TACCCCGACGATACCCAAAC-3′
	Probe	5′-/56-FAM/TGTCGAGAA/ZEN/CGCGAGCGATTCG/31ABkFQ/3′
RASSF1 [46]	Forward	5′-ATTGAGTTGCGGGAGTTGGT-3′
	Reverse	5′-ACACGCTCCAACCGAATACG-3′
	Probe	5′-/56-FAM/CCCTTCCA/ZEN/ACGCGCCA/31ABkFQ/3′
TERT [46]	Forward	5′-TGGTGATGGAGGAGGTTTAGTAAGT-3′
	Reverse	5′-AACCAATAAAACCTACTCCTCCCTTAA-3′
	Probe	5′-/56-FAM/ACCACCACC/ZEN/CAACACACAATAACAAACACA/31ABkFQ/3′

## Data Availability

The data presented in this study are available on request from the corresponding author. The data are not publicly available due to the specific condition of the Patient information leaflet that data would only be available to the study researchers and the Institution where the research took place, if necessary.
